# 

**Published:** 1888-10

**Authors:** 


					﻿Volume LVII.
OCTOBER, 1888.
I Number 4.
CHICAGO
Medical
Journal
EXAMINER
EDITOR. :
S. J. JONES, M.D., LL.D.
PUBLISHED MONTHLY UNDER THE AUSPICES OF
THE CHICAGO MEDICAL PRESS ASSOCIATION.
RAND, McNALLY & CO., PRINTERS.
Entered*! th* Po*t Office »t Chicago, for tran«mi»»ion through th* mail* *< »ec»nd-ol»** matter.
				

